# Data‐driven prediction of prolonged air leak after video‐assisted thoracoscopic surgery for lung cancer: Development and validation of machine‐learning‐based models using real‐world data through the ePath system

**DOI:** 10.1002/lrh2.10469

**Published:** 2024-10-11

**Authors:** Saori Tou, Koutarou Matsumoto, Asato Hashinokuchi, Fumihiko Kinoshita, Hideki Nakaguma, Yukio Kozuma, Rui Sugeta, Yasunobu Nohara, Takanori Yamashita, Yoshifumi Wakata, Tomoyoshi Takenaka, Kazunori Iwatani, Hidehisa Soejima, Tomoharu Yoshizumi, Naoki Nakashima, Masahiro Kamouchi

**Affiliations:** ^1^ Department of Health Care Administration and Management, Graduate School of Medical Sciences Kyushu University Fukuoka Japan; ^2^ Department of Surgery and Science, Graduate School of Medical Sciences Kyushu University Fukuoka Japan; ^3^ Institute for Medical Information Research and Analysis Saiseikai Kumamoto Hospital Kumamoto Japan; ^4^ Big Data Science and Technology, Faculty of Advanced Science and Technology Kumamoto University Kumamoto Japan; ^5^ Medical Information Center Kyushu University Hospital Fukuoka Japan; ^6^ Health Information Management Center National Hospital Organization Kyushu Medical Center Fukuoka Japan; ^7^ Division of Respiratory Surgery Saiseikai Kumamoto Hospital Kumamoto Japan; ^8^ Center for Cohort Studies, Graduate School of Medical Sciences Kyushu University Fukuoka Japan

**Keywords:** data‐driven prediction, electronic clinical pathway, lung cancer, machine learning, prolonged air leak, real‐world data, video‐assisted thoracoscopic surgery

## Abstract

**Introduction:**

The reliability of data‐driven predictions in real‐world scenarios remains uncertain. This study aimed to develop and validate a machine‐learning‐based model for predicting clinical outcomes using real‐world data from an electronic clinical pathway (ePath) system.

**Methods:**

All available data were collected from patients with lung cancer who underwent video‐assisted thoracoscopic surgery at two independent hospitals utilizing the ePath system. The primary clinical outcome of interest was prolonged air leak (PAL), defined as drainage removal more than 2 days post‐surgery. Data‐driven prediction models were developed in a cohort of 314 patients from a university hospital applying sparse linear regression models (least absolute shrinkage and selection operator, ridge, and elastic net) and decision tree ensemble models (random forest and extreme gradient boosting). Model performance was then validated in a cohort of 154 patients from a tertiary hospital using the area under the receiver operating characteristic curve (AUROC) and calibration plots.

**Results:**

To mitigate bias, variables with missing data related to PAL or those with high rates of missing data were excluded from the dataset. Fivefold cross‐validation indicated improved AUROCs when utilizing key variables, even post‐imputation of missing data. Dichotomizing continuous variables enhanced performance, particularly when fewer variables were employed in the decision tree ensemble models. Consequently, regression models incorporating seven key variables in complete case analysis demonstrated superior discriminatory ability for both internal (AUROCs: 0.77–0.84) and external cohorts (AUROCs: 0.75–0.84). These models exhibited satisfactory calibration in both cohorts.

**Conclusions:**

The data‐driven prediction model implementing the ePath system exhibited adequate performance in predicting PAL post‐video‐assisted thoracoscopic surgery, optimizing variables and considering population characteristics in a real‐world setting.

## 1 INTRODUCTION

Recent advancements in machine‐learning techniques have catalyzed a revolution across various domains, particularly in healthcare.^1^, ^2^, ^3^ A notable strength of these models lies in their ability to accurately predict future outcomes by harnessing numerous variables. Although the clinical application of machine‐learning models is expected to benefit clinicians, patients, and healthcare systems, concerns about various issues such as bias, privacy protection, and lack of transparency also exist.^1^, ^2^, ^3^ Moreover, leveraging real‐world data accumulated in daily clinical practice presents challenges. Real‐world data often lack structural uniformity and standardization, hindering their incorporation into datasets.^4^ Additionally, missing data are inevitable during routine patient care and treatment, potentially introducing significant bias.^5^ Moreover, concerns about the generalizability of machine‐learning‐based models persist as a result of the issue of overfitting. Consequently, the applicability of these techniques for predicting clinical outcomes in real‐world settings remains uncertain.

Thoracoscopic technologies have emerged as innovative surgical procedures.^6^, ^7^ Video‐assisted thoracoscopic surgery (VATS) is a widely accepted surgical modality for pulmonary resections.^8^, ^9^, ^10^ Prolonged air leak (PAL) is a common postoperative complication in patients undergoing VATS.^11^ Currently, thoracoscopic surgeons rely on their individual experience to predict the occurrence of PAL. Numerous previous studies have attempted to identify risk factors for PAL and develop predictive models. However, predicting PAL remains a challenge, and none of the models developed so far possess sufficient predictive ability.^12^, ^13^, ^14^, ^15^, ^16^, ^17^, ^18^, ^19^, ^20^, ^21^, ^22^, ^23^, ^24^, ^25^, ^26^, ^27^, ^28^ As a result, these models have not been standardized for clinical use and their clinical application has not been realized.^29^, ^30^, ^31^, ^32^ Accurate prediction would greatly assist surgeons in proactively addressing PAL in patients undergoing VATS.

To efficiently gather a wide range of variables in routine clinical practice, we introduced an electronic clinical pathway platform (or ePath system). In this study, we collected a comprehensive array of clinical variables applying the ePath system. By leveraging this dataset, machine‐learning‐based models were developed and validated for predicting PAL in patients undergoing VATS for lung cancer. With regard to machine‐learning models for tabular data, we used linear regression models such as sparse regression models with L1 and L2 regularization and decision tree ensemble learning models that capture nonlinear relationships due to their good performance. Therefore, this study aimed to determine the feasibility of data‐driven models for predicting PAL post‐VATS by utilizing comprehensive data accumulated during daily clinical practice through the ePath system.

## 2 METHODS

### 2.1 Study design and data source

The ePath system was implemented in four hospitals located in various regions of Japan (Kyushu University Hospital, Fukuoka; Saiseikai Kumamoto Hospital, Kumamoto; National Hospital Organization Shikoku Cancer Center, Matsuyama; and NTT Medical Center, Tokyo) in 2018. Detailed information regarding the system has been described previously.^33^, ^34^, ^35^ In summary, daily clinical practices are standardized through clinical pathways, with data integrated into three layers, namely, outcome, assessment, and task, including their variations. The structured data were accumulated within the ePath system.

A retrospective analysis was conducted using comprehensive data extracted through the ePath system to develop and validate data‐driven prediction models. The study protocol was approved by the Clinical Research Network Fukuoka Certified Review Board (approval no. M23082‐00). Written informed consent was obtained from all patients in the participating hospitals between July 2019 and March 2021 as well as between September 2022 and May 2023.

### 2.2 Study patients

Data from 314 consecutive patients with lung cancer treated with VATS at Kyushu University Hospital were used as the development cohort. Additionally, data from 154 consecutive patients with lung cancer who underwent VATS at Saiseikai Kumamoto Hospital were used as the validation cohort. For a complete case analysis, 279 and 114 patients were included in the development and validation cohorts, respectively, after listwise deletion of key variables (Figure S1).

### 2.3 Clinical outcome

The clinical outcome of interest was PAL post‐VATS for lung cancer. In both hospitals, chest drain removal was scheduled on postoperative day 2. Although prior studies variously defined PAL,^11^, ^36^ in this study, it was defined as chest drainage continuing on postoperative day 3 or later post‐VATS.

### 2.4 Clinical variables

Comprehensive data were extracted using the ePath system, which integrates different databases, including the Diagnosis Procedure Combination (DPC), clinical pathway, and laboratory databases stored within the hospital database infrastructure. The DPC system is a case‐mix classification procedure employed in Japan that encompasses standardized care data derived from the claims data. The DPC database contains various data types, such as Form 1 (demographic details, diagnoses, and disease severity), H files (physical status, patient care, and activities of daily living), and EF files (medications, surgeries, and other procedures). The clinical pathway database comprises outcome, assessment, and task units and variances during the care process, whereas the laboratory dataset includes blood test results obtained through the Standardized Structured Medical Information eXchange system.^11^

In total, 489 variables were collected from the derivation cohort database. Key variables were defined based on significant variances according to the PAL in the univariate analysis. To assess the impact of missing data on the outcome, the missing rates were calculated for individual variables and identified variables for which missing data did not occur completely at random as these depended on the outcome utilizing univariate analysis. When continuous data were converted into binary data, the values were dichotomized using the best threshold determined by the Youden method to discriminate PAL in the univariate analysis.

### 2.5 Dataset construction

To develop data‐driven prediction models for PAL, variables prior to and on the day of surgery were utilized as explanatory variables. Initially, the relationships between various variables and the PAL were assessed. Consequently, some variables exhibited significant variances between patients with and without PAL in the univariate analysis (Figure S2). Additionally, certain variables exhibited significant missing rates with variances depending on the PAL (Figure S3).

Prior to constructing the dataset for analysis, we investigated the relationship between the missing rates and the number of explanatory variables with rates lower than a certain threshold or the sample size in which all variables were complete (Figure S4). Consequently, the number of available variables and the sample size with complete data exhibited an inverse relationship with missing rates. As the missing rates increased, the number of available variables increased, whereas the number of patients with complete data decreased.

To construct the dataset, variables with missing rates of less than 10% were included as explanatory variables for two reasons: (1) to preserve a sufficient sample size with complete data, even with a substantial number of explanatory variables (Figure S4), and (2) to exclude variables with outcome‐related missing data (Figure S5). Therefore, 21 variables with missing rates higher than 10% and 10 variables with missing data related to PAL were all excluded. Ultimately, we used 458 variables for the development of the model (Figure S1). Among these variables, we identified 14 that significantly differed according to PAL in the univariate analysis and defined them as key variables (Figure S1).

### 2.6 Model development and validation

Prediction models were developed applying five machine‐learning algorithms: least absolute shrinkage and selection operator (LASSO),^37^ ridge,^38^ and elastic net^39^ as sparse linear regression models and random forest^40^ and extreme gradient boosting (XGBoost)^41^ as decision tree ensemble models. To develop sparse linear regression models (LASSO, ridge, and elastic net), the regularization parameter *λ* was optimized in the range of 0–1 using fivefold cross‐validation within the training data. In addition, for elastic net, the mixing parameter *α* was also optimized using the same range and procedure as those for *λ*. Similarly, for the decision‐tree‐based algorithms, the parameters were optimized using fivefold cross‐validation within the training data, just as for the sparse linear regression models. In the case of random forest, the key parameter, that is, the number of predictors used in each decision tree, was optimized in the range of 1–8. For XGBoost, in order to avoid overfitting, the optimal values for tree depth and the proportion of predictors used in each decision tree were optimized in the ranges of 3–10 and 0.8–1, respectively.

The discriminatory ability of the model was evaluated by using the area under the receiver operating characteristic curve (AUROC) and the area under the precision–recall curve. The MissForest method was utilized for imputation.^42^ Internal validation was conducted through fivefold cross‐validation, assessing discrimination through AUROCs and area under the precision–recall curves and calibration through calibration plots. Prediction models developed in the development cohort were externally validated in the validation cohort.

The significance of each variable in the sparse linear regression models (LASSO, ridge, and elastic net) was assessed by employing standardized partial regression coefficients. Variable importance was evaluated by utilizing the mean decrease accuracy in the random forest model and gain in the gradient‐boosting decision tree model.

The calibration of each model was assessed using a calibration plot. Calibration plots were obtained by plotting the predicted probabilities against the observed probabilities in the stratified risk groups estimated utilizing each predictive model. When evaluating the calibration with the external cohort, the shrinkage of the regression coefficients was conducted by applying the slope of the calibration plot.

### 2.7 Statistical analysis

Variables were compared based on PAL utilizing appropriate statistical tests, namely, *t*‐test or Mann–Whitney *U*‐test for continuous variables and chi‐squared test or Fisher's exact probability test for nominal variables. Statistical significance was defined as a two‐sided *p*‐value of <0.05. All statistical analyses were conducted using the R statistical package (http://www.r-project.org/, version 4.0.5), with detailed methods provided in Supplemental Methods.

## 3 RESULTS

### 3.1 Study patients' characteristics

**FIGURE 1:**
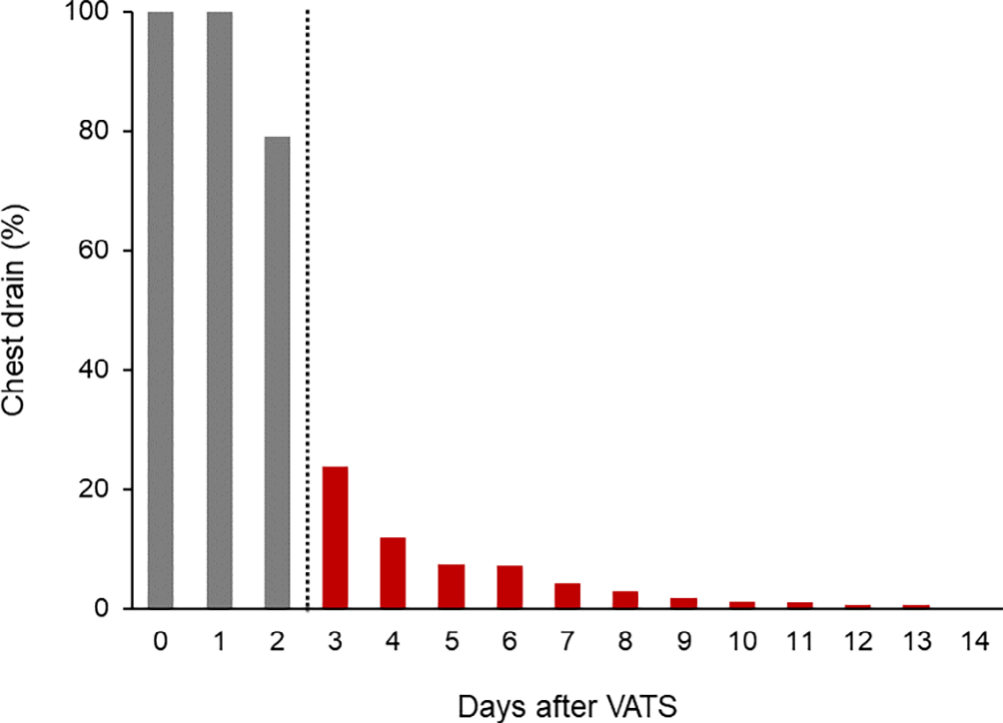
Proportion of chest drainage after video‐assisted thoracoscopic surgery (VATS). The histogram exhibits the proportion of patients requiring chest drain insertion each day post‐VATS. The electronic clinical pathway for VATS schedules drain removal on postoperative day 2.

A total of 314 patients (mean ± SD age 67.8 ± 11.7 years, male 57.3%) were included in the development cohort (Table 1). The proportion of patients in whom remaining chest drains were inserted each day post‐VATS was investigated. Chest drainage persisted for postoperative day 3 or late post‐VATS in 76 (24.2%) patients. Figure 1 illustrates the percentage of patients in whom a chest drain was inserted on each postoperative day post‐VATS.

**TABLE 1:** Baseline characteristics of patients in the development and validation cohorts.

	Development cohort	Validation cohort
	*n* = 314	*n* = 154
Age, mean ± SD	67.8 ± 11.7	70.0 ± 8.7
Male, *n* (%)	180 (57.3)	89 (57.8)
Body mass index, kg/m^2^, mean ± SD	23.4 ± 3.4	23.5 ± 3.4
Diabetes mellitus, *n* (%)	33 (10.5)	29 (18.8)
Smoking index, median (IQR)	15 (0–780)	0 (0–600)
Types of surgery, *n* (%)		
Lobectomy	140 (44.6)	119 (77.3)
Segmentectomy	51 (16.2)	7 (4.5)
Wedge resection	123 (39.2)	28 (18.2)

Abbreviation: IQR, interquartile range.

### 3.2 Model development

**FIGURE 2:**
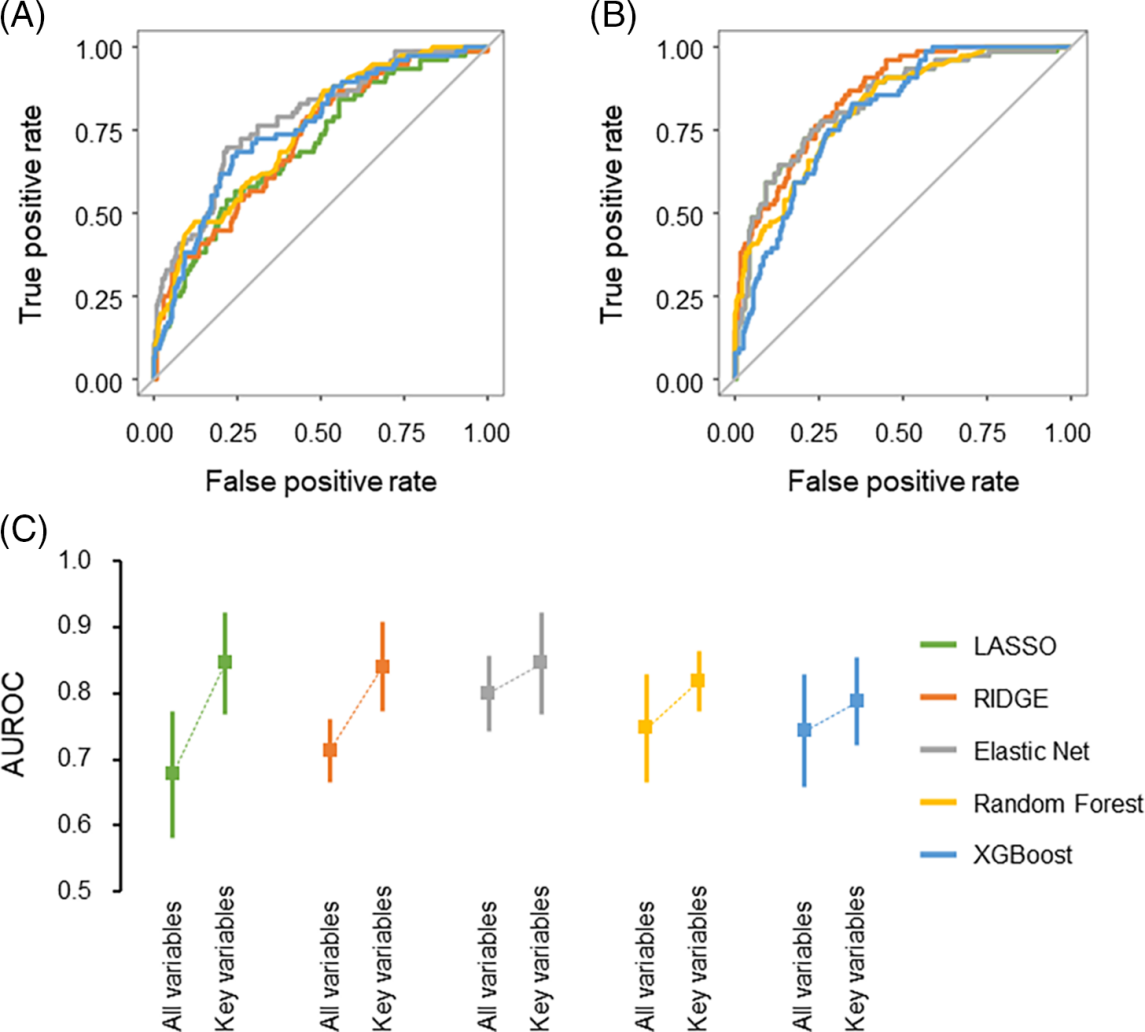
Receiver operating characteristic (ROC) curves and area under the receiver operating characteristic curves (AUROCs) in prediction models in complete case analysis. ROC curves illustrate the prolonged air leak (PAL) prediction models using all (A) or key variables (B) with complete data analysis. The ROC curves were generated through fivefold cross‐validation with the least absolute shrinkage and selection operator (LASSO) (green), ridge (orange), elastic net (gray), random forest (RF) (yellow), or extreme gradient boosting (XGBoost) (blue) models. AUROCs are displayed for PAL prediction models using all or key variables with complete data analysis (C).

**FIGURE 3:**
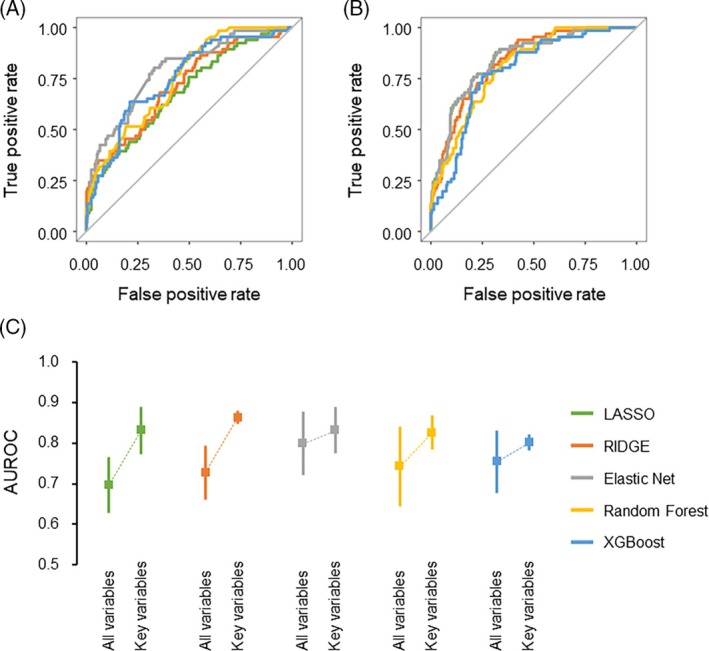
Receiver operating characteristic (ROC) curves and area under the receiver operating characteristic curves (AUROCs) in prediction models after imputing missing values. ROC curves illustrate the prolonged air leak (PAL) prediction models using all (A) or key variables (B) after imputing missing data with MissForest. ROC curves were generated through fivefold cross‐validation with the least absolute shrinkage and selection operator (LASSO) (green), ridge (orange), elastic net (gray), random forest (RF) (yellow), and extreme gradient boosting (XGBoost) (blue) models. AUROCs are displayed for the PAL prediction models using all variables or key variables after imputing missing data with MissForest (C).

Initially, a comparison was conducted between the discriminatory powers of the models constructed applying all variables (Figure 2A) and those constructed using the key variables (Figure 2B). Fivefold cross‐validation demonstrated that models utilizing key variables generally exhibited higher discriminatory power as opposed to models utilizing all of the variables (Figure 2C). When only key variables were used as explanatory variables, sparse linear regression models such as the LASSO and ridge models generally outperformed decision tree ensemble models regarding discriminatory ability (Figure 2C). The imputation method did not substantially improve the discriminatory ability (Figure 3). The AUROCs for each of the five groups during the fivefold cross‐validation, along with their means ± SD, in the development cohort are summarized in Table S1.

To ascertain the importance of the variables in each model, we assessed the significance of key variables. The 14 key variables are those that showed a significant difference depending on the presence or absence of PAL. These significant variables were selected by univariate tests, based on the presence or absence of PAL. The variable importance varied significantly between the sparse linear regression and decision tree ensemble models (Figure S6). Certain variables emerged as more significant predictive factors than others across all models. These included the use of fibrin sealant, presence of air leak on the operative day, body mass index, use of psychiatric medication, and type of surgical procedure. Continuous variables, such as body mass index, mean corpuscular hemoglobin, and smoking index, tended to be recognized as more essential in decision tree ensemble models as opposed to in sparse linear regression models.

### 3.3 External validation

**FIGURE 4:**
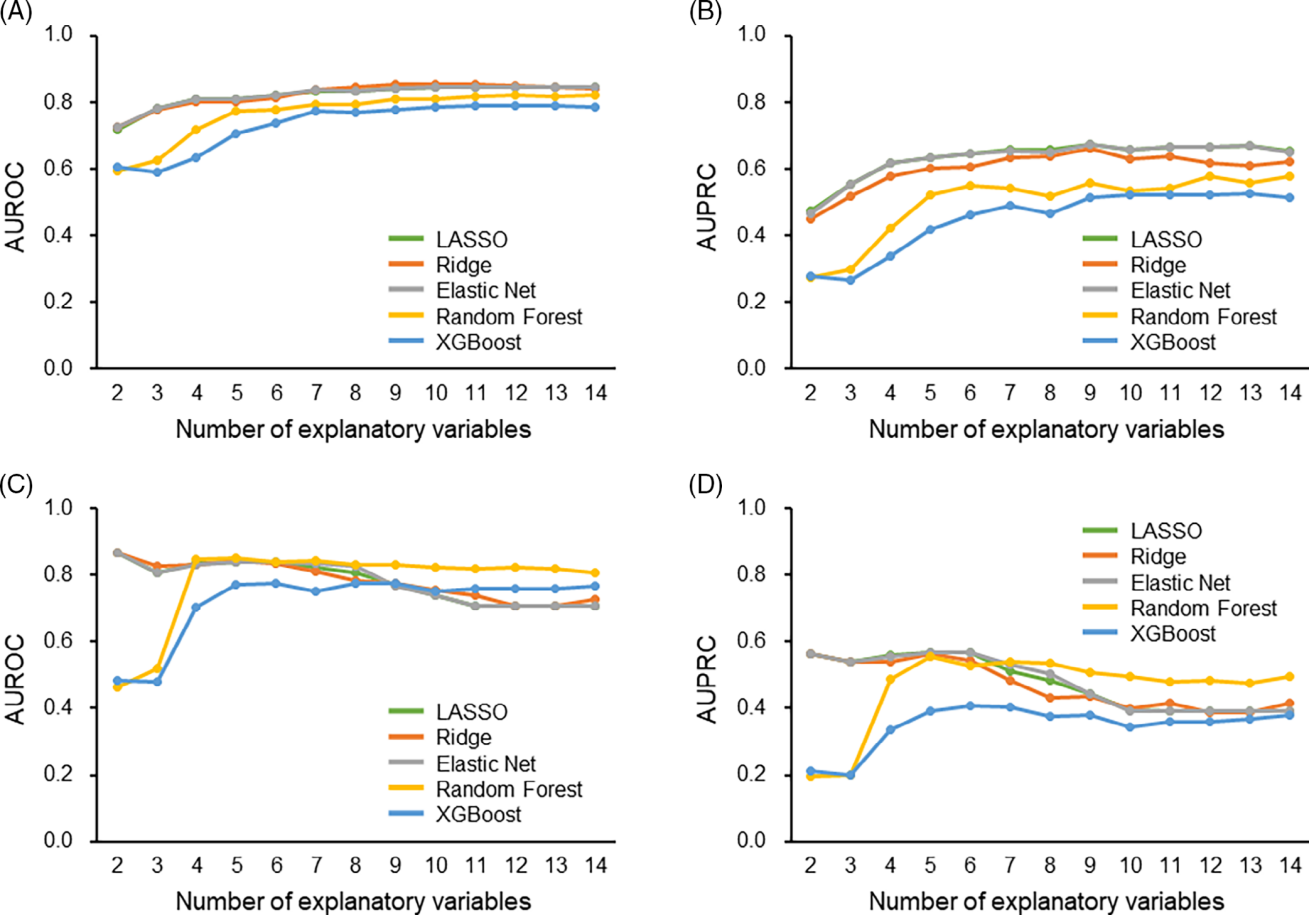
Area under the receiver operating characteristic curve (AUROCs) and area under the precision–recall curves (AUPRCs) in prediction models with varying explanatory variables. The AUROCs and AUPRCs for predicting prolonged air leak (PAL) are presented for models that utilize different numbers of variables. Changes in discriminatory power according to the explanatory variables were compared between the development ([A] AUROC; [B] AUPRC) and validation cohorts ([C] AUROC; [D] AUPRC). Calculations were based on fivefold cross‐validation after iteratively removing less important variables from all key variables applying the least absolute shrinkage and selection operator (LASSO) (green), ridge (orange), elastic net (gray), random forest (yellow), and extreme gradient boosting (XGBoost) (blue) models.

**FIGURE 5:**
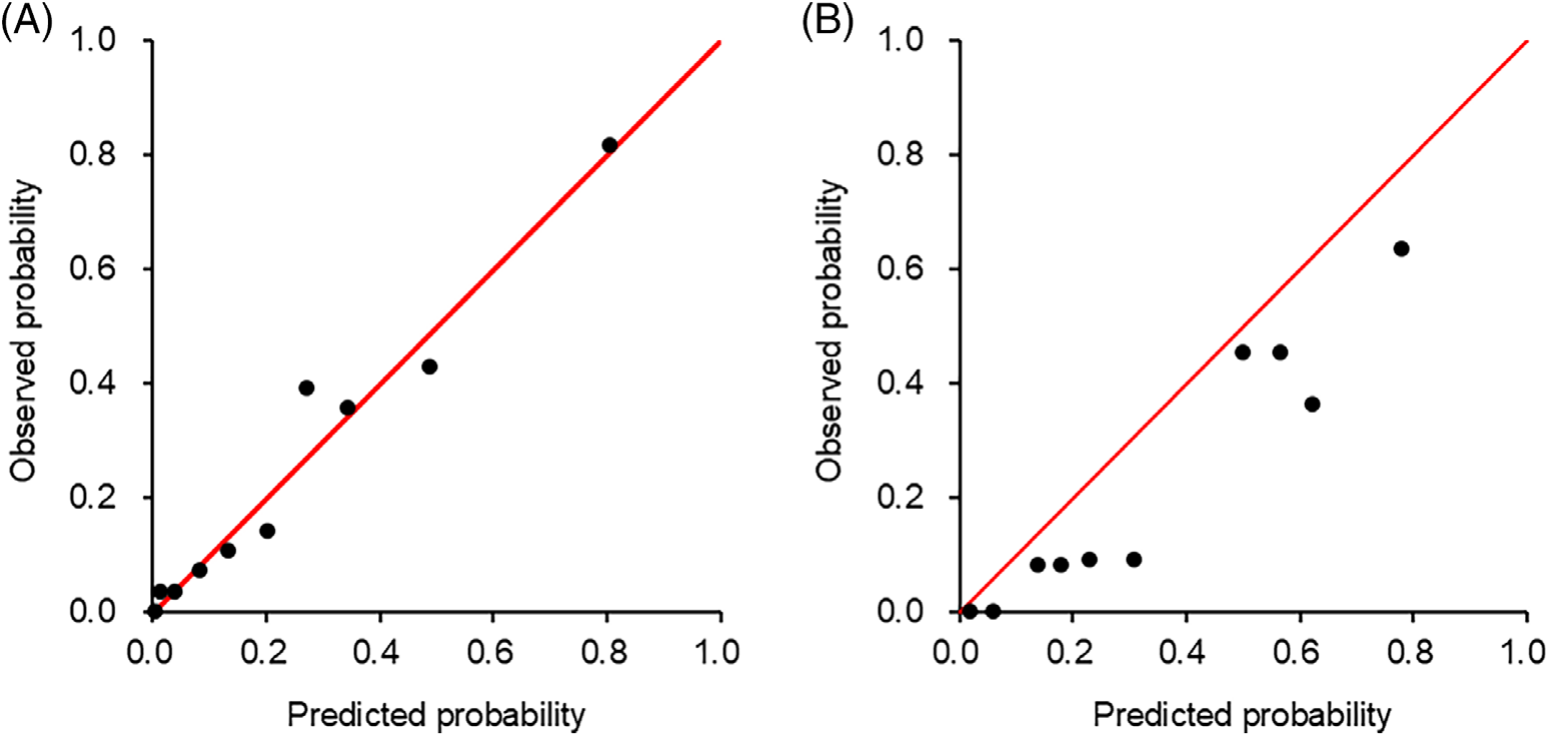
Calibration plots of the prediction model. A least absolute shrinkage and selection operator model for predicting prolonged air leak was developed using seven key variables in the development cohort (A) and validated in the validation cohort (B). The observed probabilities were plotted against the probabilities estimated utilizing the prediction model.

Subsequently, the data‐driven prediction models were externally validated in the validation cohort of 154 patients (mean ± SD age 70.0 ± 8.7 years, male 57.8%). We compared the distribution of each variable between the two patient populations from the two hospitals in Figure S7. How the model performance varied in both the development and validation cohorts was assessed when explanatory variables were sequentially excluded based on their significance (Figure 4). As the number of explanatory variables decreased, the discriminatory ability gradually worsened in the development cohort. Conversely, in the validation cohort, discrimination improved with the inclusion of several variables, but it deteriorated when only a few variables were utilized in the decision tree ensemble models (Figure 4). When the top seven key variables were included in the models, the discriminatory performance was excellent for both cohorts. Calibration plots for the prediction model with seven variables exhibited excellent concordance between the predicted and observed probabilities in the development cohort (Figure 5A). The observed probabilities increased linearly with predicted probabilities in the validation cohort (Figure 5B).

### 3.4 Impact of categorization on model performance

**FIGURE 6:**
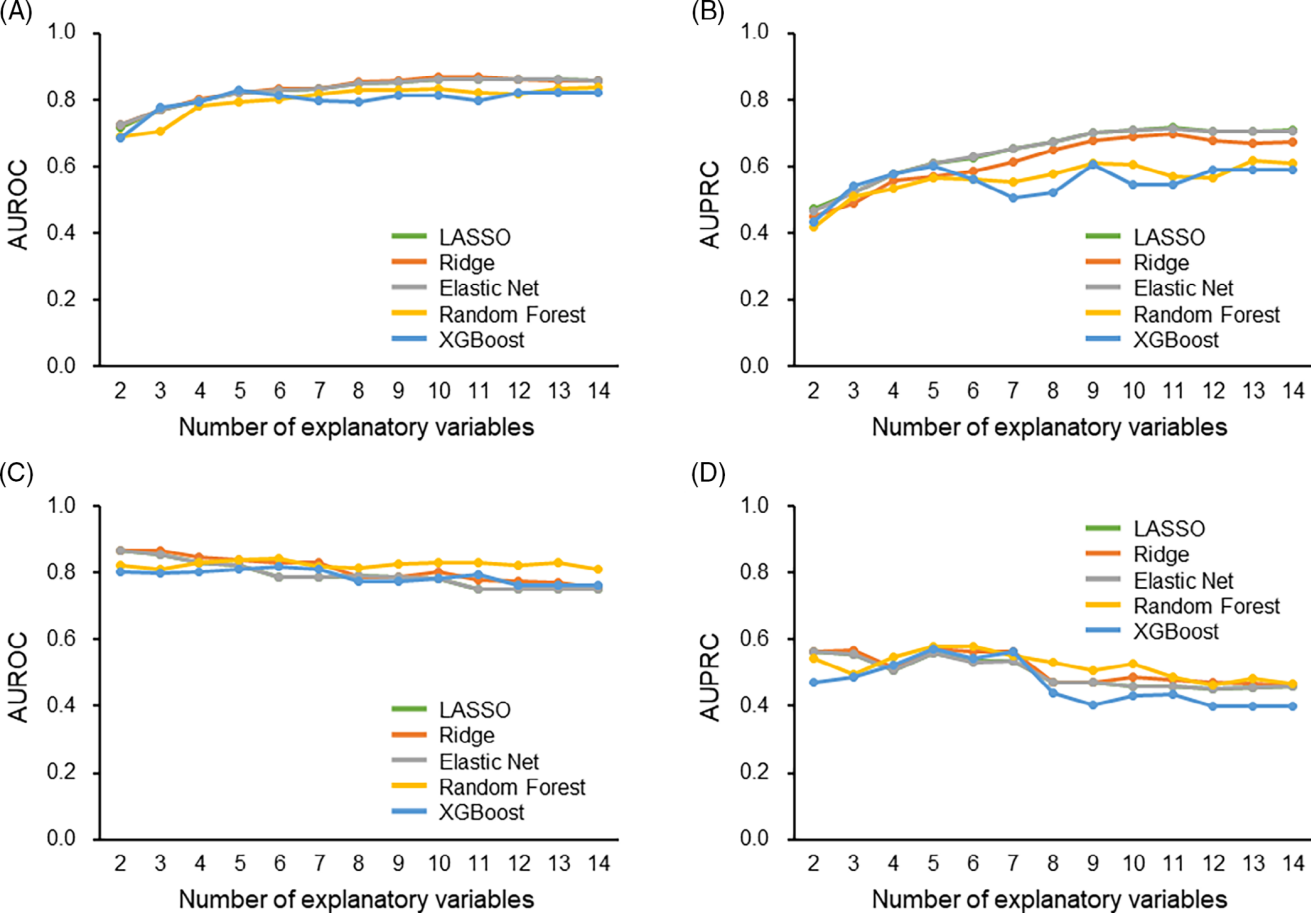
Area under the receiver operating characteristic curves (AUROCs) and area under the precision–recall curves (AUPRCs) in prediction models with varying explanatory categorized variables. The AUROCs and AUPRCs for predicting prolonged air leak (PAL) are presented for models that utilize different numbers of variables. Continuous variables were categorized into binary data with an optimal cutoff point for the PAL. Changes in discriminatory power according to the explanatory variables were compared between the development ([A] AUROC; [B] AUPRC) and validation cohorts ([C] AUROC; [D] AUPRC). Calculations were based on fivefold cross‐validation after iteratively removing less important variables from all key variables utilizing the least absolute shrinkage and selection operator (LASSO) (green), ridge (orange), elastic net (gray), random forest (yellow), and extreme gradient boosting (XGBoost) (blue) models.

**FIGURE 7:**
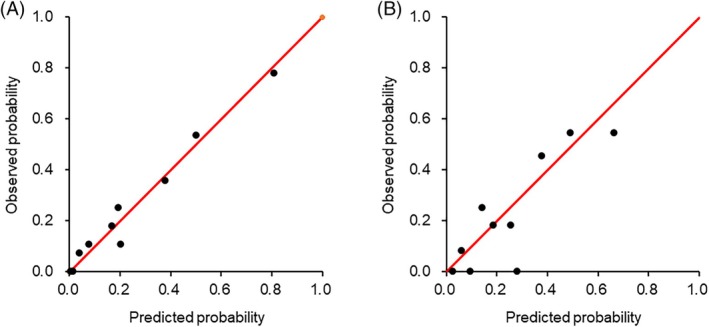
Calibration plots of prediction model utilizing categorized variables. A least absolute shrinkage and selection operator model for predicting prolonged air leak (PAL) was developed using seven key variables in the development cohort (A) and validated in the validation cohort (B). Continuous variables were dichotomized into two groups, with an optimal cutoff level for predicting PAL. The observed probabilities were plotted against the probabilities estimated utilizing the prediction model.

Given that nonlinear relationships may lead to overfitting and diminish generalization performance, particularly in decision tree ensemble models, we opted to dichotomize the continuous variables into binary data and integrate them into the model. Consequently, the variable importance of continuous data varied post‐dichotomization in the decision tree ensemble models (Figure S8). Despite the reduction in explanatory variables, the discriminatory ability was relatively high in both sparse linear regression models and decision tree ensemble models post‐dichotomization of the continuous variables into binary variables (Figure 6). Models featuring the top seven ranked categorized variables exhibited good calibration not only in the development cohort but also in the validation cohort (Figure 7).

## 4 DISCUSSION

The primary findings of this study are outlined in this section. To mitigate bias, variables with missing data pertaining to PAL, or those displaying high rates of missing data, were excluded prior to dataset construction for analysis. The discriminatory ability of the data‐driven models for predicting PAL post‐VATS for lung cancer was influenced by the selection of explanatory variables and machine‐learning algorithms. Sparse linear regression models incorporating several key predictors exhibited excellent discriminatory power for both internal and external cohorts. Dichotomizing continuous variables by using appropriate cutoff levels enhanced predictive performance, particularly in decision tree ensemble models within the external cohort. These findings indicate the feasibility of data‐driven models for predicting PAL post‐VATS for lung cancer, utilizing pertinent variables and machine‐learning algorithms when real‐world data are available through the ePath system. Optimizing variables and machine‐learning algorithms is crucial for enhancing the performance of data‐driven models regarding discrimination and calibration in real‐world scenarios.

### 4.1 Selection of explanatory variables

Several variables were extracted utilizing the ePath system. We used variance data in the clinical pathway in conjunction with claims and laboratory databases accessed through the ePath system. Among them, variables for which missing data cannot be assumed to occur completely at random may introduce serious bias when incorporated into the models. Therefore, identifying variables with missing data related to PAL is crucial to avoid bias resulting from reverse causality. Furthermore, missing data significantly affected the sample size owing to the listwise deletion in the complete case analysis. As the missing rates increased, the sample size with complete data decreased; however, the number of available variables increased. To maximize the utilization of explanatory variables, increase the sample size, and minimize bias as a result of the missing data, the optimal cutoff level for missing rates must be determined when selecting explanatory variables.

### 4.2 Key variables related to PAL

Prior studies have reported various factors associated with an increased risk of PAL after pulmonary resection utilizing open operations^12^, ^14^, ^15^, ^16^, ^17^, ^18^, ^19^, ^20^, ^22^, ^27^ or VATS.^21^, ^23^, ^25^, ^26^, ^27^, ^28^ A Manhattan plot visualizes potentially essential variables among the comprehensive data. An overview of the relationship between each variable and the outcome aids in understanding the overall predictor–outcome relationship in real‐world settings. In our machine‐learning‐based prediction models, known predictors such as sex, body mass index, smoking index, air leak on the operative day, application of fibrin sealant, surgical procedure, and steroid utilization for chronic obstructive pulmonary disease were identified as crucial predictors of PAL. Nevertheless, other factors that were not previously considered risk factors for PAL were also used in the models. For instance, the mean corpuscular hemoglobin was identified as an unfamiliar risk factor for PAL in this study. However, macrocytic anemia is often found in patients with chronic obstructive pulmonary disease.^43^, ^44^ Other factors such as comorbid diabetes, utilization of psychiatric drugs or α blockers, and the postoperative need for assistance may be related to the occurrence of PAL through biological or social mechanisms, or they reflect other factors related to PAL. Machine‐learning‐based models can enhance prediction accuracy by integrating factors that potentially contribute to PAL but have not currently been recognized as significant.

### 4.3 Development of machine‐learning‐based models

Five machine‐learning algorithms were employed to develop data‐driven models for predicting PAL post‐VATS. Fivefold cross‐validation exhibited that the selection of explanatory variables significantly affected discriminatory performance, depending on the algorithms. Restricting the variables to only key variables related to PAL in the univariate analysis drastically increased the discriminatory ability of sparse linear regression models. In contrast, the predictive performance of the XGBoost model exhibited a modest improvement after reducing the number of explanatory variables. When all available variables were included as explanatory variables, imputation of missing variables slightly enhanced discrimination performance in sparse linear regression models but resulted in little improvement in decision tree ensemble models. These findings indicate that the discriminatory ability of data‐driven prediction models is affected differently by variables and imputation depending on the machine‐learning algorithm. Hence, these characteristics must be considered by machine learning.

### 4.4 Validation of data‐driven model

The discriminatory performance varied reciprocally between the internal and external cohorts as the number of explanatory variables was sequentially reduced based on their variable importance. Internal validation revealed that discrimination remained excellent until the explanatory variables were restricted to several variables. However, a further reduction in the number of explanatory variables diminished performance. In contrast, in the external cohort, discrimination ability was enhanced but abruptly deteriorated after the restriction of the explanatory variables to a few. Therefore, a trade‐off relationship exists in the discriminatory ability between the internal and external cohorts according to the number of explanatory variables, in line with Occam's razor bounds. Variables and machine‐learning algorithms must be carefully selected to optimize the discriminatory ability in both internal and external cohorts.

Decision tree ensemble models may be advantageous for detecting nonlinear relationships in continuous variables, but overfitting may impair the generalization performance. Therefore, whether discrimination and calibration could be enhanced by dichotomizing the continuous variables into binary data was evaluated. Consequently, the discriminatory ability was excellent even in decision tree ensemble models when explanatory variables were categorized and restricted to a few key variables in both the internal and external cohorts. Therefore, categorization of variables is also important to improve the balance of predictive performance between internal and external cohorts, especially when a nonlinear relationship may be involved in the prediction.

### 4.5 Clinical relevance

Many studies have primarily used logistic models to attempt to predict PAL, but this remains a challenge. This may be due to the diversity of patients and healthcare systems. When PAL occurs, patients must endure prolonged chest tube drainage, resulting in persistent pain and requiring additional interventions, such as chemical or mechanical pleurodesis, which increase discomfort. Additionally, prolonged bed rest increases the risk of venous thrombosis and pneumonia. If an accurate prediction of PAL becomes possible, clinicians may be able to intervene early in cases of high‐risk patients.^29^, ^45^ PAL is considered to be a major cause of prolonged hospital stays, and preventing it through early intervention could lead to savings in healthcare costs, human resources, and other medical resources.^28^, ^46^, ^47^ Recently, studies have begun to apply machine‐learning models to outcomes such as length of hospital stay after VATS^48^ and PAL.^26^ Further investigation is necessary to determine whether the machine‐learning models developed in this study for PAL prediction can be applied to routine clinical practice.

A diverse spectrum of adverse events and unfavorable outcomes may develop in patients who undergo surgery. Early and accurate predictions can assist healthcare professionals in decision‐making. Machine‐learning‐based models may be promising tools for accurately predicting patient outcomes in daily clinical practice. However, challenges may arise when these models are implemented in a real‐world setting. This study demonstrated that predictive performance is highly affected by explanatory variables, study population, missing values, and machine‐learning algorithms in real‐world settings. Nevertheless, machine‐learning‐based models can be developed with excellent performance for both internal and external cohorts by appropriately controlling these factors. Developing cautious data‐driven prediction models is crucial when employing machine‐learning algorithms with comprehensive data accumulated during daily clinical practice. Further studies are required to determine the validity of data‐driven prediction models in real‐world settings.

### 4.6 Study limitations

This study presents several limitations. First, PAL was defined as delayed drain removal post‐VATS, serving as a surrogate for the event, which may potentially overestimate the actual occurrence. Second, the sample size was limited, and a larger cohort of patients might be necessary to achieve more accurate predictions of PAL post‐VATS. Third, missing data were observed for various variables, albeit the ePath system's usage frequency was relatively low. Last, the study was conducted exclusively at two hospitals in Japan, necessitating further assessment of its generalizability across other cohorts.

## 5 CONCLUSIONS

Machine‐learning‐based prediction models were developed and validated for PAL post‐VATS by utilizing real‐world data through the ePath system. By carefully controlling explanatory variables, study population, missing data, and machine‐learning algorithms, data‐driven prediction models can overcome the limitations associated with real‐world data. Leveraging methodologies facilitated by the ePath system holds promise in achieving precise data‐driven predictions in clinical practice. Such advancements could provide invaluable support to healthcare professionals, aiding them in making optimal clinical decisions tailored to individual patients.

## FUNDING INFORMATION

This study was supported by the Japan Society for the Promotion of Science (JSPS) KAKENHI (grant numbers: JP21K19648, JP22K17336, and JP23K21506).

## CONFLICT OF INTEREST STATEMENT

The authors declare they have no conflicts of interest to disclose.

## Supporting information


**Data S1.** Supporting information.
