# Supplementary material for: Data‐driven prediction of prolonged air leak after video‐assisted thoracoscopic surgery for lung cancer: Development and validation of machine‐learning‐based models using real‐world data through the ePath system
Source: Learn Health Syst. 2024 Oct 11;9(2):e10469. doi: 10.1002/lrh2.10469 (PMC12000770; doi:10.1002/lrh2.10469)
Supplement: Supplementary file 1 — Data S1. Supporting information. [file LRH2-9-e10469-s001.docx]

**Data-driven prediction of prolonged air leak after video-assisted thoracoscopic surgery for lung cancer: Development and validation of machine-learning-based models using real-world data through ePath system**

**Methods**

**Figures**

Figure S1. Study patients and variables

Figure S2. Variables associated with PAL

Figure S3. Missing data related to PAL

Figure S4. Sample size and available variables proportionate to missing rates

Figure S5. Frequency of missing values in each variable

Figure S6. Variable importance in machine-learning-based models

Figure S7. Distribution of variables in two cohorts

Figure S8. Variable importance in machine-learning-based models with categorized variables

**Tables**

Table S1. Prediction model AUROCs in five-fold cross validation in the development cohort

**Methods**

***R programs for model training process***

Data were analyzed using RStudio (http://www.rstudio.com/, version 2023.06.0) and the R statistical package (http://www.r-project.org/, version 4.0.5). For the Least Absolute Shrinkage and Selection Operator (LASSO), Ridge regression, and Elastic Net, we used the R program “glmnet” (https://cran.r-project.org/web/packages/glmnet/glmnet.pdf) and tuned the parameters using “caret” (https://cran.r-project.org/web/packages/caret/caret.pdf). The parameter λ was tuned by incrementing it from 0 to 1 in 0.2 steps, using fivefold cross-validation with grid search. For Elastic Net, the parameter α was also tuned by increasing it from 0 to 1 in 0.2 steps using a similar method. A Random Forest was conducted utilizing “randomForest” (https://cran.r-project.org/web/packages/randomForest/randomForest.pdf) and gradient boosting decision tree model using “xgboost” (https://cran.r-project.org/web/packages/xgboost/xgboost.pdf), tuning their parameters using “caret.” In the Random Forest, “mtry,” the number of variables randomly sampled as candidates for each tree split, was tuned between 1 and 8, determined by fivefold cross-validation with grid search. In the gradient boosting decision tree model, the parameters “max_depth” and “colsample_bytree” were tuned. Specifically, the tree depth was tuned to range from 3 to 10, and the proportion of randomly sampled columns for each decision tree was tuned to range from 0.8 to 1. These tuning processes were conducted using fivefold cross-validation with a grid search. Other parameters were set as follows: “nrounds” 500, “gamma” 0, “eta” 0.1, and “min_child_weight” 1. The significance of the variables was evaluated using standardized regression coefficients in the ridge regression, LASSO, and Elastic Net; mean decrease accuracy in the Random Forest model; and gain in the gradient-boosting decision-tree model.

***R programs for prediction metrics and validation***

The “pROC” (https://cran.r-project.org/web/packages/pROC/pROC.pdf) package and the “PRROC” (https://cran.r-project.org/web/packages/PRROC/PRROC.pdf) package were utilized to calculate the area under the receiver operating characteristic curve (AUROC) and the area under the precision-recall curve (AUPRC), respectively. For internal validation, the R package “rsample” (https://cran.r-project.org/web/packages/rsample/rsample.pdf) was used with a stratified fivefold cross-validation.

**Figure S1.** Study patients and variables


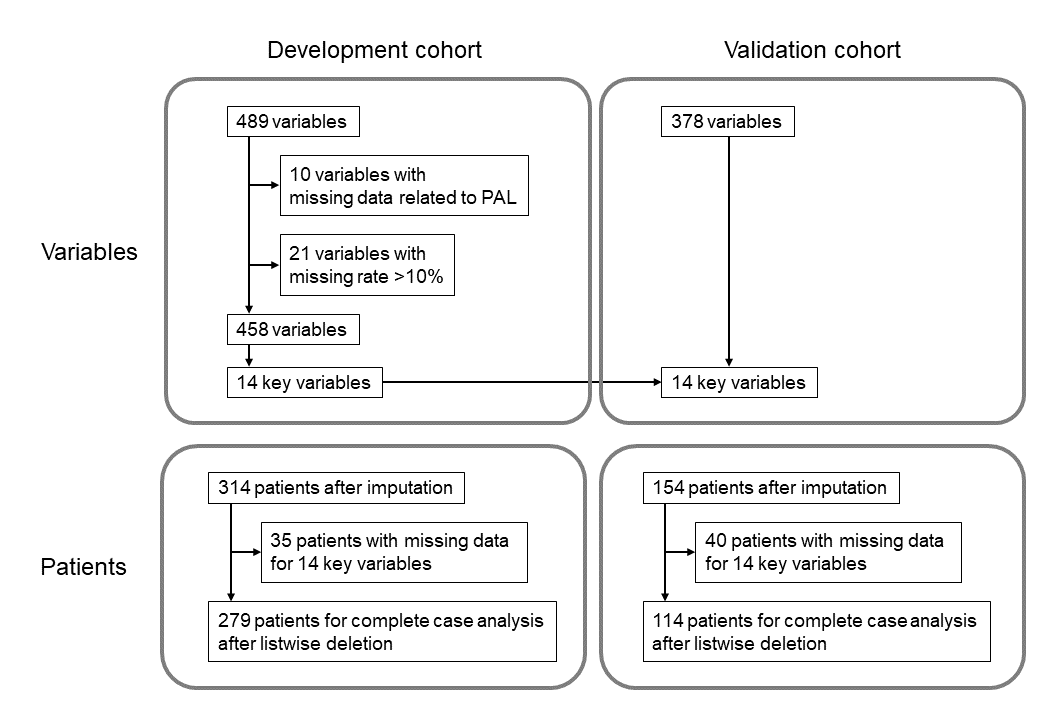


The selection of the study patients and explanatory variables are depicted in the development and validation cohorts. Among all the available variables, 458 were utilized post-exclusion of 10 variables with missing data related to PAL and 21 with missing data exceeding 10% in the development cohort. Fourteen key variables were utilized for model validation in both cohorts. In the analysis of all the cases, 314 and 154 patients were included in the development and validation cohorts, respectively, post imputation using MissForest. In the complete case analysis, 279 and 114 patients with complete data were included in the development and validation cohorts, respectively, post listwise deletion.

**Figure S2.** Variables associated with PAL


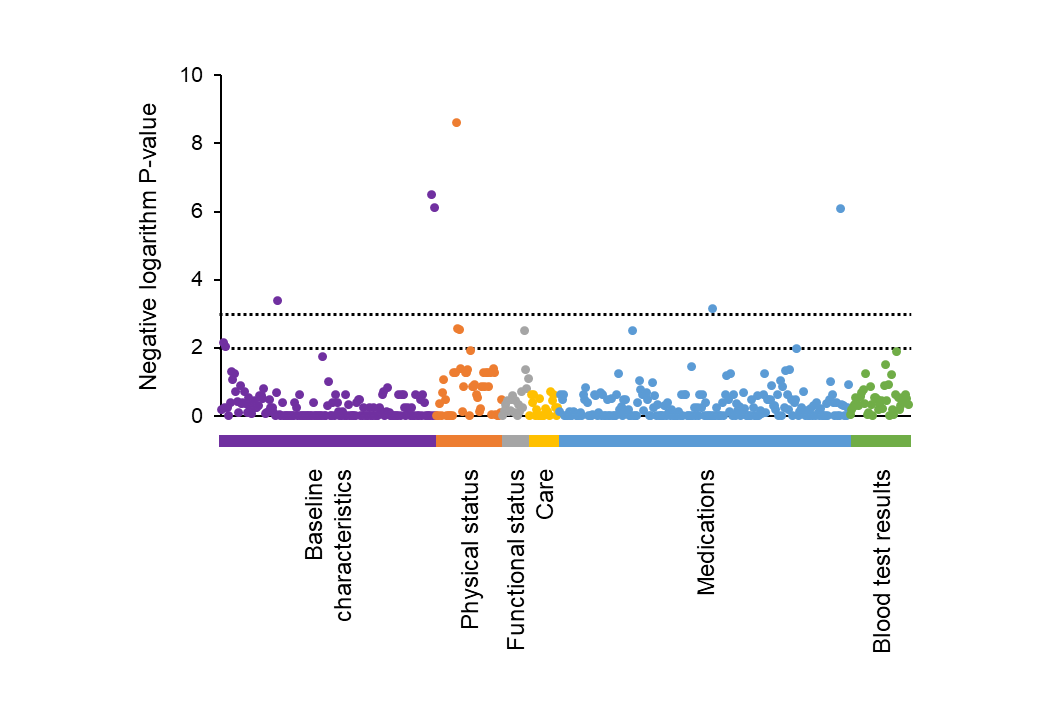


Manhattan plots depict the significance of the variances in individual PAL variables. Statistical analyses were conducted across the six variable categories. The vertical axis represents negative logarithm P-values, and the dotted horizontal lines indicate P-values of 0.01 and 0.001.

PAL: prolonged air leak.

**Figure S3.** Missing data related to PAL


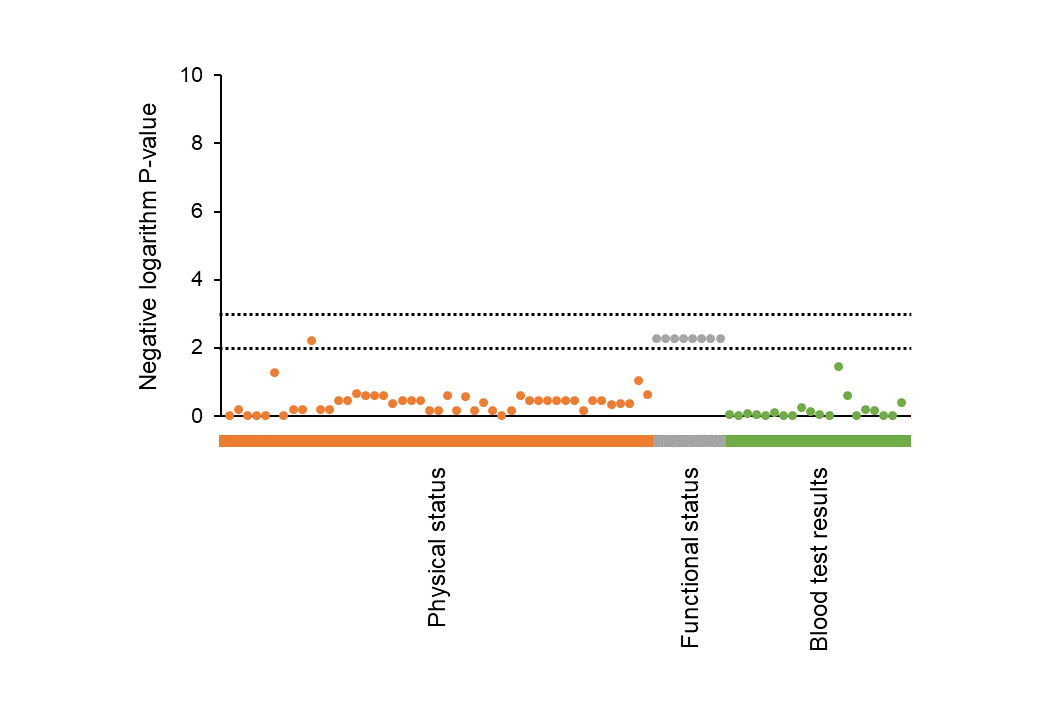


Manhattan plots demonstrate the significance of variances in missing rates among individual variables according to the PAL. Statistical analyses were conducted to determine the missing data for each variable related to PAL. The vertical axis represents negative logarithmic P-values, with dotted horizontal lines indicating P-values of 0.01 and 0.001.

PAL: prolonged air leak

**Figure S4.** Sample size and available variables proportionate to missing rates


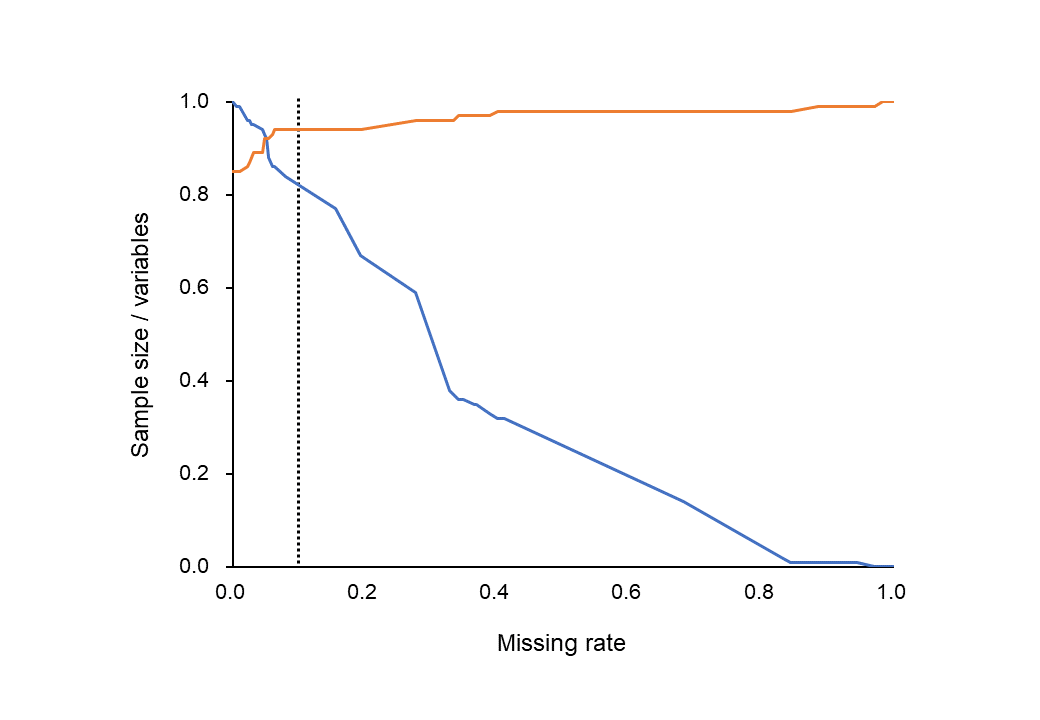


The sample size (blue line) and proportion of explanatory variables (orange line) are plotted against missing rates. The proportion of explanatory variables was calculated by dividing the number of available variables with missing rates below a certain threshold by the total number of variables. The sample size was evaluated based on the proportion of patients with complete data, when variables with a missing rate below a certain threshold were included.

**Figure S5.** Frequency of missing values in each variable


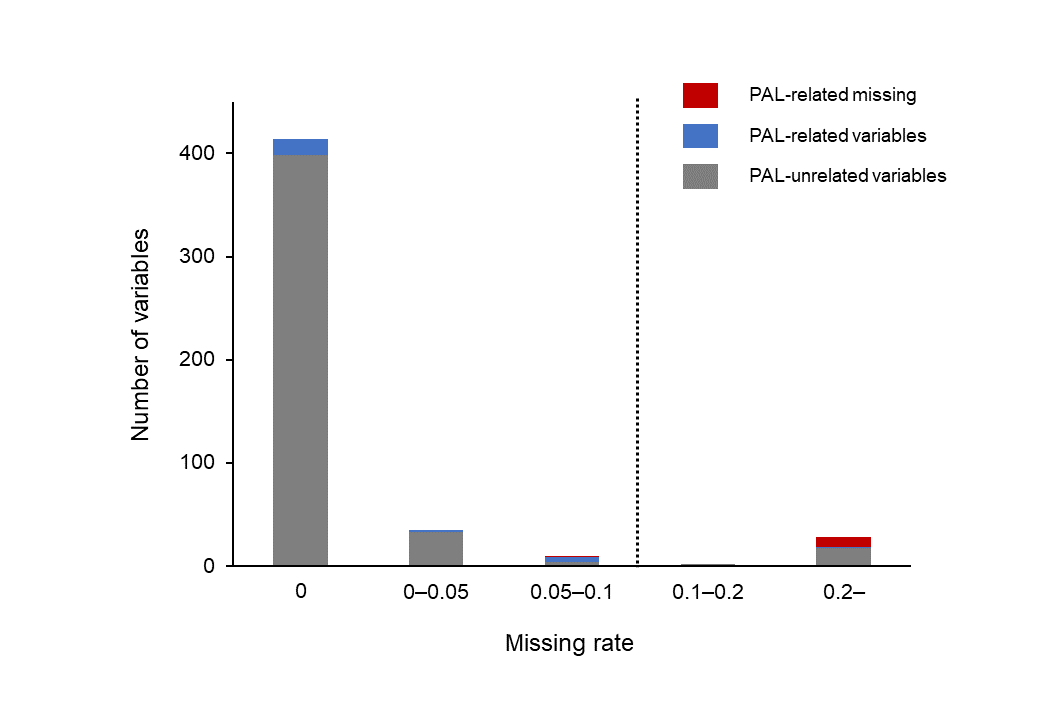


This figure exhibits the number of variables with missing data below a certain threshold. Bar graphs exhibit variables significantly differing according to PAL (blue), variables independent of PAL (gray), and variables where missing data occurred depending on PAL (red).

PAL: prolonged air leak.

**Figure S6.** Variable importance in machine-learning-based models


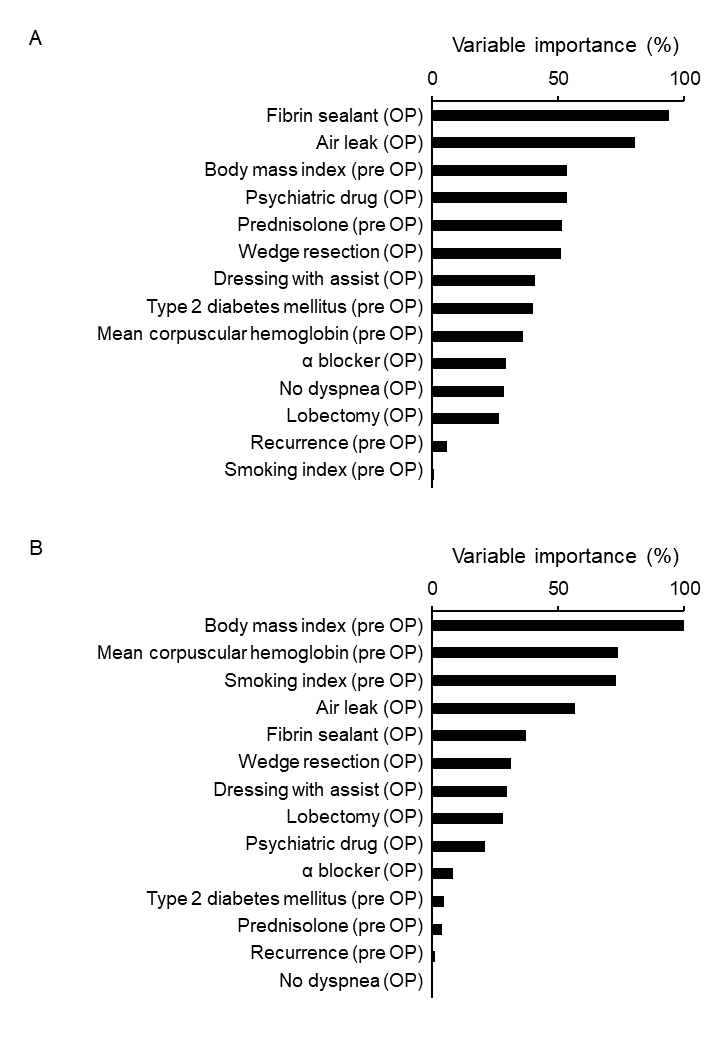


The significance of each variable is presented in the sparse linear regression models (A) and decision tree ensemble models (B). Mean values of the standardized partial regression coefficients in the LASSO, Ridge, and Elastic Net models (A) and variable importance in the Random Forest and XGBoost models (B) are expressed as a percentage of the highest value.

OP: variables on the day of surgery; preoperative OP: variables prior to surgery.

**Figure S7.** Distribution of variables in two cohorts


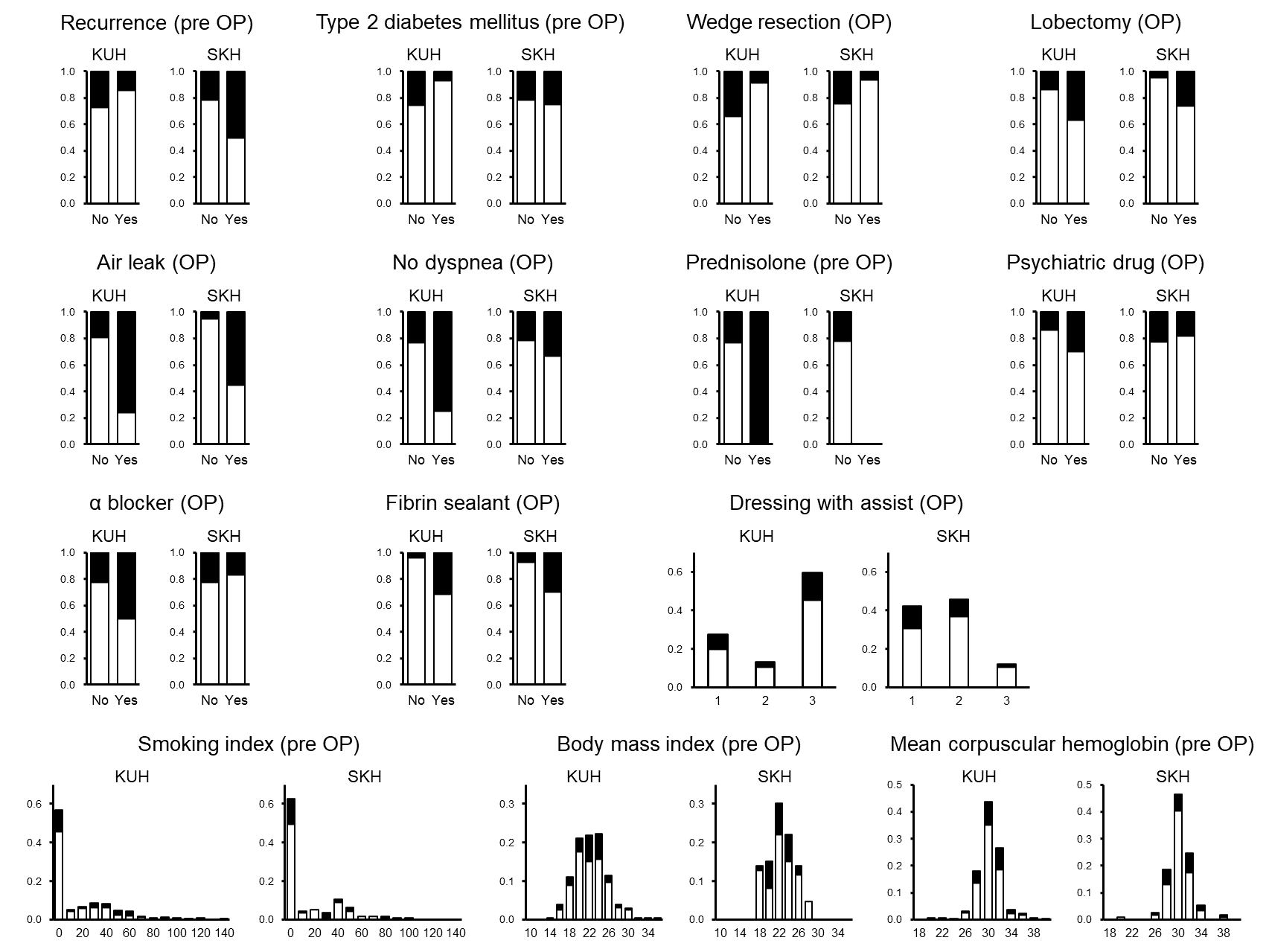


This figure shows the differences in the frequencies of prolonged air leak (PAL) between the development (Kyushu University Hospital: KUH) and validation (Saiseikai Kumamoto Hospital: SKH) cohorts for 14 key variables that were significantly different based on the presence or absence of PAL in the development cohort. For categorical variables, the bar graphs in the figure show the proportions of PAL presence (black bars) and absence (white bars) in both the No and Yes groups of each variable. For “dressing with assistance,” the figure is divided into three groups based on severity, with the proportions of PAL presence (black bars) and absence (white bars) shown for each group. Continuous variables are shown as histograms, with the proportions of PAL presence (black bars) and absence (white bars) indicated for each range. The units on the horizontal axis are smoking index (pack-year), body mass index (kg/m²), and mean corpuscular hemoglobin (pg).

pre OP: before surgery, OP: day of surgery.

**Figure S8.** Variable importance in machine-learning-based models with categorized variables


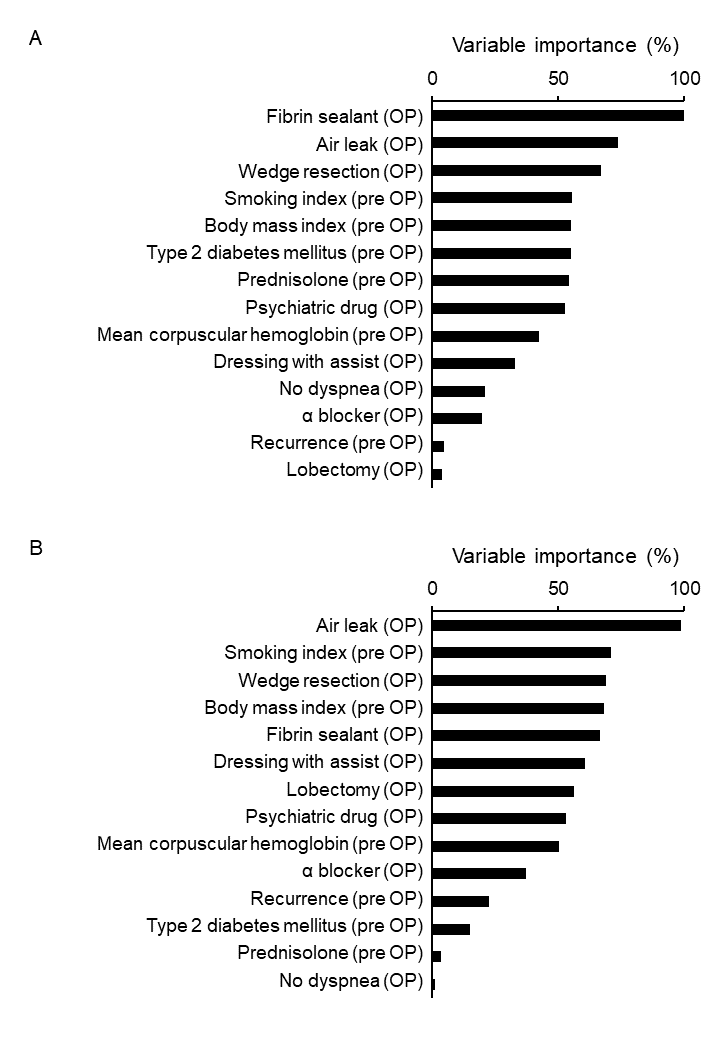


The significance of each variable post categorization is displayed in the sparse linear regression models (A) and decision tree ensemble models (B). Mean values of the standardized partial regression coefficients in the LASSO, Ridge, and Elastic Net models (A) and variable importance in the Random Forest and XGBoost models (B) are expressed as a percentage of the highest value.

OP: variables on the day of surgery; preoperative OP: variables prior to surgery.

**Table S1. Prediction model AUROCs in five-fold cross validation in the development cohort**

|  | All | G 1 | G 2 | G 3 | G 4 | G 5 |
| --- | --- | --- | --- | --- | --- | --- |
| Complete case analysis |  |  |  |  |  |  |
| All variables |  |  |  |  |  |  |
| LASSO | 0.68±0.10 | 0.60 | 0.62 | 0.71 | 0.83 | 0.63 |
| RIDGE | 0.71±0.05 | 0.69 | 0.76 | 0.64 | 0.74 | 0.73 |
| Elastic Net | 0.80±0.06 | 0.76 | 0.74 | 0.79 | 0.84 | 0.88 |
| Random Forest | 0.75±0.08 | 0.84 | 0.74 | 0.75 | 0.62 | 0.78 |
| XGBoost | 0.74±0.09 | 0.62 | 0.86 | 0.77 | 0.72 | 0.75 |
| Key variables |  |  |  |  |  |  |
| LASSO | 0.84±0.08 | 0.73 | 0.87 | 0.87 | 0.93 | 0.82 |
| RIDGE | 0.84±0.07 | 0.77 | 0.79 | 0.81 | 0.92 | 0.90 |
| Elastic Net | 0.85±0.08 | 0.87 | 0.73 | 0.82 | 0.93 | 0.88 |
| Random Forest | 0.82±0.05 | 0.76 | 0.85 | 0.85 | 0.85 | 0.78 |
| XGBoost | 0.79±0.07 | 0.81 | 0.77 | 0.84 | 0.84 | 0.68 |
| Imputed data analysis |  |  |  |  |  |  |
| All variables |  |  |  |  |  |  |
| LASSO | 0.70±0.07 | 0.68 | 0.67 | 0.65 | 0.82 | 0.67 |
| RIDGE | 0.73±0.07 | 0.76 | 0.82 | 0.73 | 0.65 | 0.68 |
| Elastic Net | 0.80±0.08 | 0.73 | 0.74 | 0.92 | 0.83 | 0.78 |
| Random Forest | 0.74±0.10 | 0.73 | 0.83 | 0.58 | 0.79 | 0.78 |
| XGBoost | 0.75±0.08 | 0.85 | 0.81 | 0.66 | 0.72 | 0.73 |
| Key variables |  |  |  |  |  |  |
| LASSO | 0.83±0.06 | 0.85 | 0.78 | 0.78 | 0.92 | 0.83 |
| RIDGE | 0.86±0.02 | 0.88 | 0.88 | 0.86 | 0.86 | 0.84 |
| Elastic Net | 0.83±0.06 | 0.78 | 0.85 | 0.83 | 0.92 | 0.78 |
| Random Forest | 0.83±0.04 | 0.88 | 0.80 | 0.82 | 0.78 | 0.85 |
| XGBoost | 0.80±0.02 | 0.80 | 0.80 | 0.83 | 0.79 | 0.78 |

AUROC: area under the receiver operating characteristic curve; PAL: prolonged air leak.

The AUROCs are shown for five groups (G1–G5), which were divided using five-fold cross-validation with the LASSO, Ridge, Elastic Net, RF, or XGBoost models. The AUROCs were calculated for PAL prediction models using all or key variables, with either complete data analysis or after imputing missing data with MissForest. The mean ± SD of AUROCs are shown for all patients.
